# Prevalence and adverse consequences of delayed diagnosis and misdiagnosis in thrombotic antiphospholipid syndrome. An observational cohort study and a review of the literature

**DOI:** 10.1007/s10067-023-06699-1

**Published:** 2023-07-15

**Authors:** Amelia Ruffatti, Marta Tonello, Antonia Calligaro, Teresa Del Ross, Maria Favaro, Margherita Zen, Ariela Hoxha, Mauro Alaibac

**Affiliations:** 1 Department of Medicine-DIMED, University Hospital of Padua, Padua, Italy; 2 Department of Medicine-DIMED, Rheumatology Unit, University Hospital of Padua, Padua, Italy; 3 Department of Medicine-DIMED, General Internal Medicine Unit, Thrombotic and Hemorrhagic Disease Unit, University Hospital of Padua, Padua, Italy; 4 Department of Medicine-DIMED, Dermatology Unit, University Hospital of Padua, Padua, Italy

**Keywords:** Adverse consequences, Antiphospholipid syndrome, Diagnostic delay, Misdiagnosis, Thrombotic antiphospholipid syndrome

## Abstract

**Obiectives:**

This study aims to prospectively evaluate the frequency and adverse consequences of diagnostic delay and misdiagnosis in a cohort of patients with thrombotic antiphospholipid syndrome (TAPS). In addition, a systematic review of the literature concerning the diagnostic delay and misdiagnosis of TAPS was carried out.

**Methods:**

Patient enrollment occurred between 1999 and 2022. The study group was formed by TAPS patients whose diagnosis was delayed and those who were misdiagnosed. The control group was made up of patients who were timely and correctly diagnosed with TAPS.

**Results:**

The literature review showed 42 misdiagnosed patients, 27 of them were in one retrospective cohort study and 15 in 13 case reports. One hundred sixty-one out of 189 patients (85.2%) received a timely, correct diagnosis of TAPS; 28 (14.8%) did not. The number of patients with diagnostic issues was significantly higher for the first period (1999–2010), and the number of patients with a correct diagnosis was significantly higher for the second one (2011–2022). When the clinical and laboratory characteristics of the patients with delayed diagnosis were compared with those with misdiagnosis, there was a significantly higher number of severe adverse consequences characterized by permanent disability or death in the latter group. The two most common types of misdiagnoses were systemic lupus erythematosus (6 cases, 46.1%) and cardiovascular diseases (4 cases, 30.8%).

**Conclusions:**

The study demonstrates that although knowledge about TAPS has improved over time, diagnostic delays and errors remains to be addressed as they are strongly associated to adverse consequences.

**Table:** 

**Key Points** *•Although knowledge of thrombotic antiphospholipid syndrome has improved over time, it is still limited.* *•Diagnostic delay and misdiagnosis are still an important issue that remains to be addressed as they are strongly associated to adverse consequences.* *•The three more frequent misdiagnoses are multiple sclerosis, systemic lupus erythematosus and cardiovascular diseases.*

## Introduction

Antiphospholipid syndrome (APS) is a hypercoagulable disorder characterized by the persistent presence in the blood of antiphospholipid antibodies (aPL) which include lupus anticoagulant (LAC) positivity and/or medium–high levels of anticardiolipin (aCL) and/or anti-beta2-glycoprotein I (anti-β2GPI) antibodies in patients with venous, arterial or micro vessels thrombosis and/or pregnancy morbidity [1]. Deep veins of the lower extremities are the most frequent sites of venous thrombosis, while the brain is the site most involved in arterial thrombosis [2]. However, atypical sites such as the abdominal aorta can also be involved [3]. In addition, APS patients are at risk for small vessel thrombosis which can be localized or spread to multiple organs [2]. APS can exist either alone or in association with other disorders, most frequently with systemic lupus erythematosus (SLE). While several proposals for the classification criteria of APS were periodically evaluated, a consensus statement was finally formulated at an international workshop held in Sapporo, Japan, in 1998 [4]. The statement defined APS as a condition that must meet at least one clinical criterion (thrombosis or pregnancy morbidity) and a laboratory one (the persistent presence of aCL antibodies and/or LAC). In conjunction with persistent anti-β2GPI antibody positivity to other laboratory tests, those two criteria were confirmed at the consensus conference held in Sydney, Australia, in 2006 [1]. For the time being, the diagnostic criteria for APS have not been defined, although the classification criteria outlined here are for the most part also used for diagnostic purposes.

APS is generally considered to fall within the group of rare diseases, being affecting ≤ 50 persons per 100,000 population [5, 6]. Recent studies have highlighted that most clinicians have only a limited knowledge about rare diseases [7, 8]. Given its rarity, patients suffering from thrombotic APS (TAPS) pose a great diagnostic challenge and are at high risk of receiving a delayed or incorrect diagnosis [9], a circumstance that can directly impact their chance of recovery and survival as they are exposed to the risk of thrombotic events with life-altering consequences during that interlude. Furthermore, a possible consequence of the delay in diagnosis can be the increase over time of the organ damage which can be determined by the damage index for thrombotic APS (DIAPS) [10, 11]. It is also important to remember that some neuropsychological or cardiac disorders, SLE or microangiopathies have clinical and/or laboratory features that overlap with those of TAPS, causing even more risk of diagnostic delay and misdiagnosis. Finally, the treatment of overlapping diseases, which is generally quite different from the antithrombotic therapy prescribed to TAPS patients, could worsen the original health problem or even cause severe harm [12, 13].

The current study presents a literature review as well as an evaluation of the frequency of diagnostic delay and misdiagnosis occurring in a cohort of TAPS patients followed up prospectively. The adverse consequences linked to delays and errors are also examined.

## Materials and Methods

### Literature review

In accordance with the Preferred Reporting Items for Systematic Reviews and Meta-Analyses (PRISMA) checklist protocol [14], a systematic review of full text manuscripts in the English language was carried out. Studies focusing on the diagnostic delay and misdiagnosis of TAPS with or without pregnancy morbidity published between July 1999 (the time that the Sapporo updated classification criteria of TAPS were published) and November 2022 were included in the search. The diagnostic delay due to delayed diagnosis or misdiagnosis was defined as the time between the onset of clinical manifestations suggestive of TAPS and the formulation of a correct diagnosis. The papers exclusively regarding purely obstetric APS were excluded from the study. Two authors (AR and MT) reviewed the literature and made the final decision independently and blindly. The records were retrieved by searching Medline via Pubmed, Scopus and Web of Science Databases. The references of relevant articles were also hand-searched to identify other potentially relevant studies. The online search was limited to observational studies (cohort, case–control and case series studies), but given the rarity of TAPS, the search strategy also included case reports. Positioned in different combinations in order to improve the sensitivity of the search strategy, the keywords entered into the search engine were: thrombotic antiphospholipid syndrome, antiphospholipid syndrome, primary antiphospholipid syndrome, secondary antiphospholipid syndrome, antiphospholipid syndrome related to other diseases, diagnostic delay, misdiagnosis and adverse consequences. The titles and abstracts of the articles originally identified were screened and those needing further examination were pinpointed. Once that phase was completed, all of the full-text articles identified were evaluated and the studies eligible for inclusion were determined.

### Study population

Study group: was formed by the patients with onset of clinical manifestations suggestive for TAPS between July 1999 and November 2022. The inclusion criteria were the following: detection in outpatient clinic of the Rheumatology Unit of the Padua University Hospital of diagnostic issues such as the delay of a TAPS diagnosis or a misdiagnosis in patients with the clinical and laboratory classification criteria for TAPS as established by Sapporo or Sydney Consensus Conferences [1, 4]. The adverse consequences of the diagnostic delay or of the misdiagnosis were identified and registered. Both in patients with diagnostic delay and in those with misdiagnosis the time between the first manifestation and the correct TAPS diagnosis always was greater than six months.

Control group: included patients in whom the onset of clinical manifestations of TAPS occurred between July 1999 and November 2022, who received timely a correct diagnosis of TAPS according to the clinical and laboratory criteria formulated during the Sapporo or Sydney consensus conferences [1, 4]. In these patients the interval between the first manifestation and TAPS correct diagnosis varied between 3 and 6 months, the time necessary to have the confirmation of aPL positivity and to perform adequate blood tests and instrumental examinations.

### Autoantibody detection

ACL and anti-β2GPI antibodies were determined by ELISA assays using a home-made method described elsewhere [15]. ACL antibody values were expressed in IgG phospholipid (GPL) and IgM phospholipid (MPL) units, respectively. The results of anti-β2GPI antibodies were expressed in arbitrary units. The cut-off values for the medium–high levels of aCL and anti-β2GPI antibodies were calculated as > the 99^th^ percentile. LAC was assessed using a three-step procedure carried out utilizing platelet-poor plasma samples following updated guidelines and utilizing diluted Russell Viper Venom and diluted Activated Partial Thromboplastin Times as screening tests [16].

### Statistical analysis

The categorical variables were expressed as frequencies and percentages; the continuous variables were expressed as mean and standard deviation. Univariate analysis was performed to evaluate the association between the categorical variables using Fischer’s exact test, and between the continuous variables using a nonparametric Mann–Whitney *U* test. A < 0.05 *p* value was considered significant. All statistical analyses were performed using GraphPad Prism statistical software (San Diego, CA, USA).

## Results

### Literature Review

**Fig. 1:**
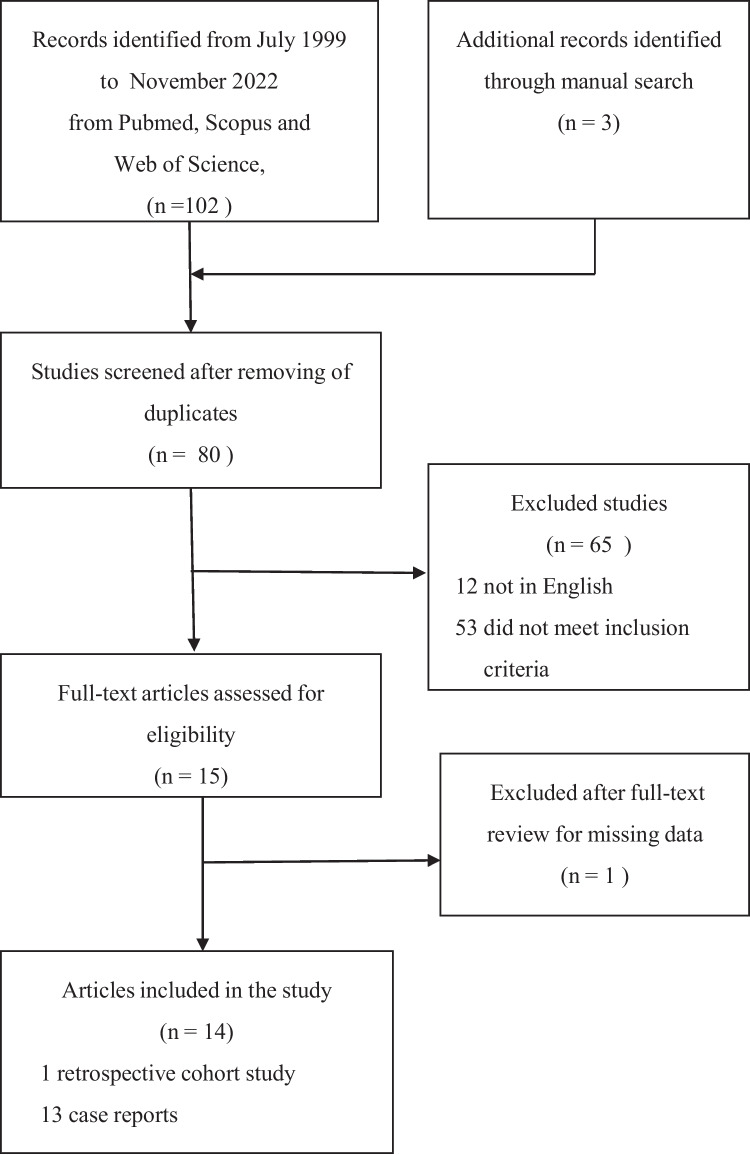
Flowchart showing the article selection process

As outlined in Fig. 1, 14 observational articles reporting data regarding TAPS misdiagnosis were found. These included 1 retrospective cohort study [17] and 13 case reports [18–30]. Overall, during the 1999 to 2022 period, 42 cases of TAPS misdiagnosis were described. Table 1 shows the clinical and laboratory characteristics of the patients at the time they were misdiagnosed and at the time the correct diagnosis of TAPS was formulated. Crucially, the most frequent incorrect diagnoses formulated were for: multiple sclerosis (29, 69.0%), cardiovascular diseases (6, 14.3%) and SLE (4, 9.5%). Thirty-seven misdiagnoses (88.1%) were done during the first period (1999–2010), and five (11.9%) during the second one (2011–2022); there was a significant difference in the numbers referring to the two periods (p = 0.0001). At the time of misdiagnosis aPL were not tested in 11 cases (26.2%). Twenty-five patients (59.5%) developed adverse consequences linked to the misdiagnosis, which led to a permanent disability or death in 14 (56.0%) of them. The final, correct diagnoses were: primary TAPS in 27 (64.3%) patients and TAPS secondary to SLE in 15 (35.7%).

**Table 1:** List of the observational studies published between 1999 and 2022 focusing on the misdiagnosis of thrombotic antiphospholipid syndrome

[Ref] (year)	Cases’ number	Clinical manifestations suggestive of APS	aPL at misdiagnosis	Misdiagnosis	Adverse consequences	aPL profile at APS diagnosis	APS diagnosis
[18] 2000	First Second	Thickening of a mitral valve leaflet, dilatative cardiomyopathy Skin lesions, livedo reticularis	IgG aCL IgG aCL	SLE SLE	Death. Autopsy: thrombotic microangiopathy of kidney, heart, brain, skin Death. Autopsy: thrombotic microangiopathy of lung, pleura, heart, brain, skin	Not reported Not reported	Catastrophic APS and SLE Catastrophic APS and SLE
[19] 2000	1	Sensory impairment, spastic gait, dysarthria thrombocytopenia	IgG aCL	Thrombotic thrombocytopenic purpura in SLE	Acral purpuric lesions, paresis in the extremities, involuntary movements, hyp esthesia, renal failure. Death	LAC and IgG aCL	Catastrofic APS (autopsy diagnosis) in SLE
[17] 2000	27	Livedo reticularis 1/27, pulmonary embolism and DVT 4/27	IgG aCL 20/25 IgM aCL 21/25 LAC 7/21	Multiple sclerosis in everyone	Livedo reticularis 4/27, migraine 3/27, splinter hemorrhages 2/27, cardiac valve disease 2/27	IgG aCL 20/25 IgM aCL 21/25 LAC:7/21	Primary APS 16/27 APS and SLE 11/27
[20] 2001	1	Hyperintense lesions in the brain, thrombocytopenia,	IgM aCL	Dystonia-parkinsonism	No clinical improvement following conventional therapy	IgG and IgM aCL	Primary APS
[21] 2001	1	Large nodules on both mitral and aortic leaflets	aPL not tested	Rheumatic valve disease	Mitral stenosis and regurgitation, aortic regurgitation	Anti-β2GPI	Primary APS with polyvalvar disease
[22] 2003	1	Cardiac prosthetic valve thrombosis	aPL not tested	Culture-negative endocarditis	Tricuspid and mitral regurgitations, mitral stenosis	LAC, IgG aCL	Primary APS with polyvalvar disease
[23] 2006	First Second	Right hemiparesis 4/5 and hemihypoesthesia Facial nerve and right lower limb palsies	aPL not tested IgG aCL	Multiple sclerosis Multiple sclerosis	Right hemiparesis and hemihypoesthesia Left hemiparesis, tactile hypoesthesia	IgM aCL IgM aCL	Primary APS Primary APS
[24] 2008	1	Retinal thrombosis, ischemic changes in glomerular basement membrane	IgG aCL and IgM aCL	Vasculitis associated to parvovirus B19 infection	Heart and renal failure, livedo reticularis, ischemic lesions of the toes	IgG aCL and IgM aCL	Catastrofic APS with parvovirus B19 infection as triggering
[25] 2008	1	Ischemic lesions on the feet, thrombocytopenia	aPL not tested	SLE	Splenic and renal infarcts, ischemic pancreatitis, retinopathy	LAC	APS and SLE
[26] 2012	1	Persistent fever and hypoxemia	aPL not tested	Pneumonia	Pulmonary embolism	IgG aCL	Primary APS
[27] 2015	1	Dyspnea, Evans syndrome, DVT of lower extremities, ischemic lesions of left foot	aPL not tested	Severe asthma	Inferior vena cava renal veins, common iliac veins, common femoral veins DVT and pulmonary thromboembolism	IgG aCL and IgM anti-β2GPI	Primary APS
[28] 2015	1	Mobile mass in the right atrium, thrombocytopenia,	aPL not tested	Right atrial myxoma	Resection of the right atrial mass, tricuspid valve ring annuloplasty	LAC	Primary APS with a thrombus in the right atrium,
[29] 2019	1	Pulmonary embolism, right atrial mass, thrombocytopenia	aPL not tested	Right atrial myxoma	Excision of right atrial mass, tricuspid valve replacement	LAC, IgG and IgM aCL, IgG and IgM anti-β2GPI	Primary APS with a calcified right atrial thrombus
[30] 2021	1	Ischemic lesions of fingers and toes, thrombocytopenia, mild renal failure	aPL not tested	Thromboangiitis obliterans (Buerger's disease)	Hemorrhagic alveolitis, renal thrombotic microangiopathy, amputations of fingers and toes	LAC	Catastrofic APS

*Ref* reference, *APS* antiphospholipid syndrome, *aPL* antiphospholipid antibodies, *IgG aCL i*mmunoglobulin G anticardiolipin antibodies, *SLE* systemic lupus erythematosus, *LAC* lupus anticoagulant, *DVT* deep vein thrombosis, *IgM aCL* immunoglobulin M anticardiolipin antibodies*, IgG anti-β2GPI* immunoglobulin G anti-β2Glycoprotein I antibodies, *IgM anti-β2GPI* immunoglobulin M anti-β2Glycoprotein I antibodies

No observational studies concerning a diagnostic delay were identified. However, there was an Italian Regional Rare Disease Registry referring to 740 patients with a definite diagnosis of APS characterized by thrombosis and/or pregnancy morbidity, registered between 1983 and 2015, where a mean diagnostic delay of 4.7 years ± 8.3 SD was reported; the figure resulted significantly reduced over time when data were stratified by time period [9].

### Our cohort’s data

Between July 1999 and November 2022, 189 patients attending the rheumatology outpatient clinic were ultimately diagnosed with TAPS. One hundred and thirty-eight were women (73.0%) and 51 (27.0%) men; the mean age at the time they were diagnosed was 46.0 years ± 13.9 SD. A timely and correct diagnosis of TAPS, in accordance with the Sapporo or Sidney classification criteria [1, 4], was formulated for 161 (85.2%) of them. A diagnostic issue was registered for 28 (14.8%) of the patients; 15 of them (7.9%) had a diagnostic delay and 13 (6.9%) a misdiagnosis. The clinical and laboratory characteristics of the patients who received a correct diagnosis and those who received a tardy diagnosis or were misdiagnosed are outlined in Table 2. Data analysis showed that there was a significantly higher percentage of females in the correctly diagnosed TAPS group and a higher percentage of males in the group with diagnostic issues. It also showed that there was a significantly higher number of patients with diagnostic issues in the first period, and a significantly higher number of patients with correct TAPS diagnosis in the second one. Finally, it was found that in a significantly high number of patients with diagnostic issues, the determination of aPL at the onset of the clinical manifestations was missing. The correctly diagnosed patients and those with diagnostic issues did not show any significant differences in the type of vascular involvement and aPL antibody profile at onset or in the clinical form of TAPS.

**Table 2:** The clinical and laboratory characteristics of patients who received a timely diagnosis of thrombotic antiphospholipid syndrome and of those with diagnostic issues

Clinical and laboratory characteristics	APS patients with timely diagnosis n.161	APS patients with diagnostic issues n. 28	Statistical comparison p =
Mean age (years) at the onset (± SD)	45.3 (13.9)	49.7 (13.1)	0.1559
Gender n (%)			
women	123 (76.4)	15 (53.6)	*0.0195
men	38 (23.6)	13 (46.4)	*0.0195
Years of the onset n (%)			
1999–2010	67 (41.6)	20 (71.4)	*0.0040
2011–2022	94 (58.4)	8 (28.6)	*0.0040
First vascular involvement n (%)			
Arteries	60 (37.3)	6 (21.4)	0.1335
Veins	55 (34.2)	9 (32.1)	1.0000
Microcirculation	17 (10.5)	7 (25.0)	0.0582
Associations	27 (16.8)	2 (7.1)	0.2613
not specified	2 (1.2)	4 (14.3)	*0.0048
Antiphospholipid antibodies not tested at the onset n (%)	0	14 (50)	*0.0001
Antiphospholipid antibody profiles at APS diagnosis n (%)			
LAC	9 (5.6)	3 (10.7)	0.3913
IgG/IgM aCL	4 (2.5)	0	1.0000
IgG/IgM aβ2GPI	6 (3.7)	0	0.5943
IgG/IgM aCL + LAC	6 (3.7)	2 (7.1)	0.3372
IgG/IgM aβ2GPI + LAC	0	0	-
IgG/IgM aCL + IgG/IgM aβ2GPI	26 (16.1)	5 (17.9)	0.7858
IgG/IgM aCL + IgG/IgM β2GPI + LAC	110 (68.3)	18 (64.3)	0.6670
Clinical forms of APS n (%)			
primary	129 (80.1)	24 (85.7)	0.6081
secondary	32 (19.9)	4 (14.3)	0.6081

*APS* antiphospholipid syndrome, *LAC* lupus anticoagulant, *IgG/IgM aCL* immunoglobulin G/M anticardiolipin antibodies, *IgG/IgM anti-β2GPI* immunoglobulin G/M anti-β2Glycoprotein I antibodies

^*^significant value

The clinical and laboratory characteristics of patients with delayed diagnosis or misdiagnosis are outlined in Table 3 and 4, respectively. The adverse consequences caused by the diagnostic issues and the outcome after the correct diagnosis of TAPS are also outlined. In both groups there was a higher frequency of diagnostic errors between the 1999–2010 period with respect to the 2011–2022 one, a high number/percentage of patients who were not tested for aPL antibodies, and a long delay during which the patients in both groups developed adverse consequences before the correct diagnosis was made. In patients with diagnostic issues the most frequent first clinical manifestation that led to a diagnostic delay or misdiagnosis was deep vein thrombosis (DVT) of lower or upper limbs, present in 40% and 23.1% of the cases, respectively; while, the first manifestations leading to the correct TAPS diagnosis was an adverse consequence of diagnostic delay. In particular, in both patients with diagnostic delay and in those with misdiagnosis the most frequent manifestation leading to correct diagnosis was arterial thrombosis found in 40% and 61.5% of cases, respectively and characterized mainly by ischemic stroke. These manifestations were reported in detail in Table 3 and 4, respectively. Even in patients with timely and correct diagnosis the most frequent first manifestation of TAPS which led to the diagnosis was arterial thrombosis present in 37.3% of cases (Table 2). In patients with timely and correct TAPS diagnosis aPL were tested at the diagnosis time (Table 2). While in both groups with delayed diagnosis and misdiagnosis aPL were determined at the first clinical manifestations and re-tested after the occurrence of adverse consequences at the time of correct TAPS diagnosis (Tables 3 and 4). The medical consultations of patients with timely and correct diagnosis were on average three, a number certainly much lower than that of patients with diagnostic delay or misdiagnosis. Unfortunately, it was not possible to make a statistical comparison because most of these latter patients came to our tertiary center after a large, but unspecified number of medical consultations made by doctors of other centers. Importantly, there were no thrombotic events after the correct diagnosis of TAPS was made and the appropriate antithrombotic treatment started. The main misdiagnoses formulated were: SLE in 6 cases (46.1%) and cardiovascular diseases in 4 (30.8%). The characteristics of the two groups are compared in Table 5: the TAPS patients who were misdiagnosed were significantly younger with respect to the patients with a delayed diagnosis. Furthermore, there was a significantly higher percentage of severe adverse consequences characterized by permanent disability or death in the patients who were misdiagnosed. There were two deaths: a patient diagnosed with SLE in whom the diagnosis of secondary TAPS was formulated 8 years late (Table 4, case 7) who died at the age of 47 of sepsis due to severe ischemic lesions in the inferior and superior limbs and a patient who was diagnosed with a mitral valve infectious endocarditis who died at the age 52 of catastrophic APS (Table 4, case 10). As far as the clinical forms of TAPS were concerned, there was a significantly higher percentage of primary TAPS in the patients with a delayed diagnosis, and there was a higher percentage of secondary TAPS in the misdiagnosed patients (as SLE in 75% of the cases). There were no significant differences between the patients with delayed diagnosis and those with misdiagnosis as far as the mean age at the time of onset of manifestations suggestive of TAPS, the type of vascular involvement, the aPL antibody profile, the failure to order aPL antibody testing and the mean delay in the TAPS correct diagnosis were concerned.

**Table 3:** The clinical and laboratory features of patients who received a delayed diagnosis of thrombotic antiphospholipid syndrome between 1999 and 2022

ID	Sex	Date at onset year	Age at onset years	Clinical manifestations at onset	aPL at onset	Delay in diagnosis years	Adverse consequences	APS diagnosis year	aPL at diagnosis	Outcome after APS diagnosis
1	M	2004	50	Pulmonary thromboembolism	aPL not tested	16	Ischemic stroke	Primary APS 2020	LAC, IgG aCL, IgG anti-β2GPI	Improvement, no further thrombosis
2	F	2005	58	Right lower limb DVT	aPL not tested	7	Recurring DVP	Primary APS 2012	LAC, IgG/IgM aCL, IgG/IgM anti-β2GPI	No further thrombosis
3	M	2005	46	Thrombosis left renal artery	LAC, IgG aCL, IgG anti-β2GPI	9	Left hand finger ischemia	Primary APS 2014	LAC, IgG aCL, IgG anti-β2GPI	Improvement, no further thrombosis
4	F	2006	40	Myocardial infarction, dizziness, scotoma	aPL not tested	11	Recurrent ischemic strokes	Primary APS 2017	IgG aCL, IgG anti-β2GPI	Improvement, no further thrombosis
5	M	2007	24	Acral ischemic lesions of upper and lower limbs, TIA	aPL not tested	5	CAPS (brain, kidney, heart, retina and skin)	Primary APS 2012	LAC, IgG/IgM aCL, IgG/IgM anti-β2GPI	Improvement, no further thrombosis
6	F	2007	32	Ischemic stroke	aPL not tested	10	Bilateral renal microinfarcts	Primary APS 2017	LAC, IgG aCL, IgG anti-β2GPI	No further thrombosis
7	M	2009	58	Left twin, popliteal and posterior tibial, right perforating DVT	aPL not tested	10	Right tibial and popliteal DVT, cerebral ischemic areas	Primary APS 2019	IgM aCL, IgM anti-β2GPI	No further thrombosis
8	F	2009	29	Left lower limb DVT, right retinal thrombosis with eye visual loss	aPL not tested	7	Left lower limb DVT, floating thrombus in the right atrium, CAPS during puerperium	Primary APS 2016	LAC, IgG aCL, IgG anti-β2GPI	Improvement, no further thrombosis
9	F	2010	41	Right retinal artery thrombosis	IgM aCL	7	Left retinal artery and vein thrombosis	Primary APS 2017	IgM aCL, IgG anti-β2GPI	No further thrombosis
10	F	2013	39	Left jugular, subclavian, axillary and brachial veins DVT	LAC	9	Left femoral, popliteal and right subpopliteal veins DVT, pulmonary embolism	Primary APS and lupus- like syndrome 2022	LAC	No further thrombosis
11	M	2015	53	Aortic thrombosis, right femoropopliteal DVT, pulmonary embolism	aPL not tested	1	Ischemic stroke	Primary APS 2016	IgG aCL, IgG anti-β2GPI	Improvement, no further thrombosis
12	F	2016	55	TIA	LAC, IgG aCL, IgG anti-β2GPI	6	Great saphenous vein thrombosis	Primary APS 2022	LAC, IgG aCL, IgG anti-β2GPI	No further thrombosis
13	M	2017	71	Pulmonary thromboembolism, right femoropopliteal DVT	aPL not tested	4	Ischemic stroke,	Primary APS 2021	LAC	Improvement, no further thrombosis
14	F	2019	60	Acute myocardial infarction without epicardial coronary stenoses	aPL not tested	2	Left hemisome paresthesia, dizziness, ischemic areas in the brain	Primary APS 2021	LAC	Improvement, no further thrombosis
15	M	2019	43	Renal failure stage III, hypertension	LAC, IgG aCL, IgG anti-β2GPI	2	Mitral valve disease, ischemic areas in the brain	Primary APS 2021	LAC, IgG aCL, IgG anti-β2GPI	Improvement, no further thrombosis

*ID* identification number*, APS* antiphospholipid syndrome*, aPL* antiphospholipid antibodies, *M* male, *F* female, *TIA* transient ischemic attack, *CAPS* catastrofic antiphospholipid syndrome, *LAC* lupus anticoagulant*, IgG aCL* immunoglobulin G anticardiolipin antibodies, *IgM aCL* immunoglobulin M anticardiolipin antibodies, *IgG anti-β2GPI* immunoglobulin G anti-β2Glycoprotein I antibodies, *IgM anti-β2GPI* immunoglobulin M anti-β2Glycoprotein I antibodies, *DVT* deep vein thrombosis

**Table 4:** The clinical and laboratory features of misdiagnosed thrombotic antiphospholipid syndrome patients between 1999 and 2022

ID	Sex	Misdiagnosis year	Age at misdiagnosis years	Clinical manifestations at onset	aPL at onset	Adverse consequences	APS diagnosis year	aPL at diagnosis	Outcome after APS diagnosis
1	M	SLE 1999	36	Skin ischemic lesions	LAC, IgG aCL, IgG anti-β2GPI	Myocardial infarction without epicardial coronary stenoses	APS and lupus-like syndrome 2014	LAC, IgG aCL, IgG anti-β2GPI	No further thrombosis
2	F	SLE 2000	28	Finger and toe ischemia, phosphenes	IgG aCL,	Ischemic stroke with left hemiparesis	Primary APS 2008	LAC, IgG aCL, IgG anti-β2GPI	Improvement, no further thrombosis
3	F	Systemic sclerosis 2000	30	Right lower limb DVT	LAC, IgG/ IgM aCL, IgG/IgM anti-β2GPI	Recurrent ischemic strokes	APS and systemic sclerosis 2010	LAC, IgG aCL, IgG anti-β2GPI	Improvement, no further thrombosis
4	F	Polychon- dritis 2002	52	Pulmonary thromboembolism	IgG aCL	Recurrent pulmonary thromboembolism	Primary APS 2017	LAC, IgG aCL, IgG anti-β2GPI	No further thrombosis
5	F	SLE 2003	21	Right big toe ischemia	LAC, IgM aCL	Popliteal artery thrombosis, toes’ischemia with amputation	APS and SLE 2005	LAC, IgM aCL,	Improvement, no further thrombosis
6	M	Behçet's disease 2004	35	Retinal thrombosis, diplopia, dizziness	aPL not tested	TIA, decreased strength in the lower limbs	Primary APS 2021	LAC, IgM aCL, IgM anti-β2GPI	Improvement, no further thrombosis
7	M	SLE 2005	36	Thrombosis of the arterio-venous fistula for hemodialysis, hemorrhagic alveolitis, two TIA,	LAC, IgG aCL, IgG anti-β2GPI	Aortic regurgitation with replacement, myocardial infarction, arterio-venous fistula rethrombosis, ischemic lesions, amputations of four toes and one finger. Death for sepsis	APS and SLE 2013	LAC, IgG aCL, IgG anti-β2GPI	-
8	M	SLE 2005	31	Renal failure, renal transplantation, right femoral and-popliteal DVT	LAC, IgG aCL	Mitral valve disease, loss of the transplanted kidney, ischemic stroke, epilepsy, right popliteal DVT	Primary APS 2017	LAC, IgG aCL	No further thrombosis
9	M	Acute coronary syndrome 2007	25	Acute myocardial infarction	aPL not tested	Relapsing acute myocardial infarction	Primary APS 2011	LAC, IgG aCL, IgG anti-β2GPI	No further thrombosis
10	M	Mitral valve infectious endocarditis 2008	43	Mitral valve disease, left hemiparesis	aPL not tested	Multiorgan failure with multiple infarcts in the lungs and kidneys. Death	Catastrofic APS in primary APS 2017	LAC, IgG/IgM aCL, IgG/ IgM anti-β2GPI	-
11	F	Blue toe syndrome 2010	38	Recurring TIA, ischemic necrosis of the fifth left toe, ischemic stroke	IgG aCL	Myocardial infarction, ischemic lesions of the fingers	Primary APS 2014	LAC, IgG/IgM aCL, IgG/IgM anti-β2GPI	Improvement, no further thrombosis
12	F	SLE 2012	41	Epilepsy with cerebral ischemic microlesions	LAC, IgG/ IgM aCL, IgG/IgM anti-β2GPI	Microinfarcts of the spleen and both kidneys	APS and SLE 2014	LAC, IgG/ IgM aCL, IgG/IgM anti-β2GPI	No further thrombosis
13	F	Paraneo- plastic syndrome 2019	63	DVT of the right twin and popliteal veins	aPL not tested	Recurrent ischemic stroke, splenic infarction	Primary APS 2020	IgG/M aCL, IgM anti-β2GPI	Improvement, no further thrombosis

I*D* identification number, *APS* antiphospholipid syndrome*, aPL* antiphospholipid antibodies, *M* male*, F* female, *SLE* systemic lupus erithematosus*, IgG aCL* immunoglobulin G anticardiolipin antibodies, *LAC* lupus anticoagulant, *IgG anti-β2GPI* immunoglobulin G anti-β2Glycoprotein I antibodies, *IgM aCL* immunoglobulin M anticardiolipin antibodies*, DVT* deep vein thrombosis*, IgM anti-β2GPI* immunoglobulin M anti-β2Glycoprotein I antibodies*, TIA* transient ischemic attack

**Table 5:** The clinical and laboratory characteristics of patients who received a delayed diagnosis of thrombotic antiphospholipid syndrome and those who were misdiagnosed

Clinical and laboratory characteristics	APS patients with delayed diagnosis n 15	APS patients with misdiagnosis n 13	Statistical comparison p =
Mean age (years) at the onset (± SD)	46.6 (12.9)	36.8 (11.3)	*0.0426
Gender n (%)			
women	8 (53.3)	7 (53.8)	1.0000
men	7 (46.7)	6 (46.1)	1.0000
Years of the onset n (%)			
1999–2010	9 (60)	11 (84.6)	0.2213
2011–2022	6 (40.0)	2 (15.4)	0.2213
Vascular involvement at the onset n (%)			
arteries	5 (33.3)	1 (7.7)	0.1727
veins	6 (40.0)	3 (23.1)	0.4348
microcirculation	2 (13.3)	4 (30.8)	0.3720
associations	1 (6.7)	2 (15.4)	0.0691
not specified	1 (6.7)	3 (23.1)	0.3111
Antiphospholipid antibody profiles at APS diagnosis n (%)			
LAC	3 (20.0)	0	0.2262
IgG/IgM aCL	0	0	-
IgG/IgM aβ2GPI	0	0	-
IgG/IgM aCL + LAC	0	2 (15.4)	0.2063
IgG/IgM aβ2GPI + LAC	0	0	-
IgG/IgM aCL + IgG/IgM aβ2GPI	4 (26.7)	1 (7.7)	0.3333
IgG/IgM aCL + IgG/IgM β2GPI + LAC	8 (53.3)	10 (76.9)	0.2543
Antiphospholipid antibodies not tested at the onset n (%)	10 (66.7)	4 (30.8)	0.1283
Mean delay (years) in APS diagnosis (± SD)	7.1 (4.0)	7.5 (4.7)	0.8896
Adverse consequences n (%)	15 (100)	13 (100)	1.0000
Permanent disability or death n (%)	6 (40.0)	11 (84.6)	*0.0238
Clinical forms of APS n (%)			
primary	15 (100)	9 (69.2)	*0.0349
secondary	0	4 (30.8)	*0.0349

*APS* antiphospholipid syndrome, *LAC* lupus anticoagulant, *IgG/IgM aCL* immunoglobulin G/M anticardiolipin antibodies, *IgG/IgM anti-β2GPI* immunoglobulin G/M anti-β2Glycoprotein I antibodies

^*^significant value

## Discussion

This is the first observational cohort study to evaluate the frequency and adverse consequences of delayed diagnosis and misdiagnosis in TAPS patients. According to data from the literature cases [17–30] and the Italian Regional Rare Disease Registry [9], the number of patients with diagnostic issues was significantly higher over the first part of the study period (1999–2010), and the number of patients with a correct diagnosis was significantly higher in the second one (2011–2022). The increasing availability of medical information and the easier access to diagnostic tests over time could have apparently contributed to preparing clinicians to address the challenge of recognizing TAPS patients.

Despite the lower frequency of TAPS in the males, there was a significantly higher percentage of men in the group of patients with diagnostic issues with respect to those who were correctly diagnosed. The result could have some explanations: as autoimmune diseases affect mainly females, the differential diagnosis with autoimmune conditions is less obvious when dealing with male patients; on the other hand, cardiovascular diseases are more common among males, thus physicians could underestimate other possible differential diagnoses, including TAPS. As there were no significant differences in the age, type of vascular involvement, aPL antibody profile, and the clinical form of TAPS between the correctly diagnosed patients and those with diagnostic issues, presumably the delays or misdiagnoses were linked to an inadequate preparation of clinicians as far as TAPS is concerned. The hypothesis is consistent with the finding of a significant high number of patients with diagnostic issues who were not tested for aPL antibodies at the onset of clinical manifestations (Table 2). It is also important to note that in clinical practice the manifestations suggestive of TAPS including venous, arterial or micro vessel thrombosis can also be indicative of other more frequently observed disorders such as cardiovascular diseases, hypertension, diabetes, SLE or other systemic autoimmune diseases, therefore it could be difficult for several doctors attributing them to a rare and little known disease such as TAPS and activating the diagnostic workup also including the determination of the aPL antibodies.

In this experience the clinical manifestation that most frequently led to the diagnostic issues was DVT both in patients with diagnostic delay and in those with misdiagnosis. DVT is a disease that can be due to several disorders such as neoplasms, haematological diseases or discoagulopathies, which are commonly found in the general population and which could mislead the doctor from the diagnosis of a rare disease such as TAPS. Indeed, in 66.7% of these patients with DVT aPL determination was not performed. Instead, the most frequent adverse consequence that led both patients with diagnostic delay and those with misdiagnosis to the correct diagnosis was arterial thrombosis and mainly ischemic stroke, a severe disease that requires an in-depth diagnostic procedure that also includes aPL testing.

An analysis of the study’s data uncovered a long time lag between the onset of clinical manifestations and the correct diagnosis of TAPS in both the patients with delayed diagnosis and in those who were misdiagnosed (Table 5); this could explain the adverse consequences found in all the patients with diagnostic issues. Just as in those cases reported in the literature (Table 1), our TAPS patients who were misdiagnosed with SLE (Table 4, cases 1, 2, 5, 7) or with cardiovascular diseases (Table 4, cases 6, 9, 10, 11) suffered from more severe adverse consequences associated to permanent disability or death. Notably the secondary form of TAPS was found only in the misdiagnosed patients; in 75% of this group of patients TAPS was associated with SLE, which presumably delayed diagnosis and treatment.

In accordance with the cases reported in the literature, the diagnoses frequently formulated prior to a TAPS diagnosis were SLE [18, 19, 25] and cardiovascular diseases [21, 22, 24, 28–30], which were recorded overall in 76.9% of our patients. Although originally described in connection to SLE, APS was recognized as a primary disease in the late 1980s [31]. Since APS and SLE share several clinical and immunological features such as hematological, cardiac, renal and neurological manifestations as well as aPL antibodies, it can be quite challenging to distinguish between the two disorders. In a recent study [32] the Systemic Lupus International Collaborating Clinics’ (SLICC) classification system for SLE [33] was assessed in a cohort of 100 patients with primary APS. The study found that 28% of the patients could have been mistakenly classified as SLE. Although the new American College of Rheumatology/European League Against Rheumatism (ACR/EULAR) 2019 classification criteria [34] have not been tested in a primary APS setting, Signorelli et al. [35] reported that the ACR/EULAR 2019 criteria [34] had a higher accuracy with respect to the SLICC 2012 one [33] in differentiating primary TAPS from SLE in 67 patients (misclassification 6.0% *vs* 35.8%). Another factor that may contribute in clinical practice to the tendency to mistake TAPS for SLE could be lupus anticoagulant's misleading name that could cause inexperienced physicians to lean towards a diagnosis of SLE, a more frequent and better known disease. As has been reported in the literature [18, 19, 25, 32] and observed in our study, the TAPS patients who were diagnosed with SLE were prescribed inappropriate treatments such as corticosteroids and/or immunosuppressive drugs and not the life-saving anticoagulant and/or antiplatelet treatments they necessitated (Table 4, cases 1, 2, 5, 7, 8, 12). As is well known, the heart is a target organ in TAPS. Although not included in the current classification criteria, heart valve disease is considered one of the most frequent cardiac manifestations in patients with TAPS [13, 36]. The disorder is easily misdiagnosed as rheumatic valve disease or infectious/culture-negative endocarditis. Crucially, early diagnosis and aggressive anticoagulation treatment are considered imperative to avoid thromboembolism, further valvular destruction and/or myocardial dysfunction in these patients [13, 36].

Limitations of the study: our experience was gained exclusively in a rheumatological context, therefore it mainly includes patients with misdiagnosis of TAPS as SLE or cardiovascular diseases. It seems probable that a not well defined percent of patients diagnosed with multiple sclerosis do in fact have APS, a condition with a totally different treatment and prognosis. Indeed, the clinical presentation and lesions evidenced by magnetic resonance imaging may be similar and therefore lead to a misdiagnosis [37]. However, a misdiagnosis of multiple sclerosis is manly reported in the literature by immunological and neurological centers [17, 23, 38]. Furthermore, until 2005 the diagnosis of TAPS was made according to the Sapporo classification criteria published in 1999 [4], which required the repeated detection of aCL and/or LAC and not of anti-β2GPI antibodies; the latter was subsequently included in the Sydney criteria published in 2006 [1]. Therefore, between 1999 and 2005, TAPS patients who were positive only for anti-β2GPI antibodies were not diagnosed correctly.

Our data demonstrate that although more knowledge about TAPS, a rare, life-threatening disease, has become available, diagnostic delays and misdiagnoses continue to be associated to adverse consequences. What have we learned from this study? The findings suggest that medical schools need to train practitioners and specialized physicians to recognize the symptoms and manifestations of TAPS in order to be able to order the diagnostic tests (LAC, aCL and anti-β2GPI antibodies) to detect the syndrome. Physicians need to pay special attention to distinguishing TAPS from its overlappers, such as multiple sclerosis, SLE and cardiovascular diseases. It would also be important that the ACR/EULAR task forces improve the specificity and sensitivity of the current classification criteria by making them more detailed especially with regard to the type of organ damage that characterizes thrombosis in TAPS, something that would help physicians recognize and diagnose TAPS. They could also include other clinical manifestations now considered non-criteria such as nephropathy and cardiac valvulopathy in the ongoing classification criteria of TAPS and recommend the use of new laboratory tests such as anti-prothrombin-phosphatidylserine antibodies [39, 40]. A correct, timely diagnosis and appropriate treatment can make an important difference in saving lives and improving patients’ prognoses.

## Data Availability

The datasets generated during and/or analysed during the current study are available from the corresponding author on reasonable request.
